# Gut Microbiota Dysbiosis Drives and Implies Novel Therapeutic Strategies for Diabetes Mellitus and Related Metabolic Diseases

**DOI:** 10.3389/fimmu.2017.01882

**Published:** 2017-12-20

**Authors:** Xuan Li, Keita Watanabe, Ikuo Kimura

**Affiliations:** ^1^Department of Applied Biological Science, Graduate School of Agriculture, Tokyo University of Agriculture and Technology, Fuchu-shi, Japan; ^2^AMED-CREST, Japan Agency for Medical Research and Development, Tokyo, Japan

**Keywords:** gut microbiota dysbiosis, metabolic diseases, immune responses, nutrient-sensing receptors, energy metabolism

## Abstract

Accumulating evidence over the past decade has linked the development of metabolic syndrome related to diabetes to variations in gut microbiota, an emerging, critical homeostatic regulator of host energy metabolism and immune responses. Mechanistic studies in rodent models have revealed an ever-increasing multitude of molecular mechanisms whereby the gut microbiota interacts with various host sensing and signaling pathways, leading to modulation of endocrine system, immune responses, nervous system activity, and hence, the predisposition to metabolic diseases. Remarkably, the microbiota-driven immune responses in metabolic tissues and the host nutrient-sensing mechanisms of gut microbial metabolites, in particular short-chain fatty acids, have been significantly associated with the proneness to diabetes and related disorders. This review will synthesize the recent efforts on unraveling the mediating role of gut microbiota in the pathogenesis of metabolic diseases, aiming to reveal new therapeutic opportunities.

## Diet-Driven Gut Microbiota Dysbiosis is Causally Linked to the Type II Diabetes Mellitus (T2DM) and Related Metabolic Disorders

Type II diabetes mellitus, a major constituent of metabolic diseases, is clinically hallmarked by hyperglycemia, impaired peripheral response to insulin (i.e., insulin resistance) and pancreatic β-cell decompensation. T2DM is essentially linked to obesity and the pathophysiology of both entails facets of gene–diet/lifestyle interactions. Obesity and T2DM also increase the incidence of non-alcoholic steatohepatitis and cardiovascular disease. Gut microbiota is a highly complex bacterial community that indigenously colonizes the gastrointestinal tract. It constitutes such a tremendous pool of microbial species and genetic variability that the alternations in its composition and repertoire of metabolites and components trigger markedly diverse host responses. To date, a wealth of evidence has emerged, among which a remarkable example is the replication of obese phenotypes of human discordant twin donors in rodent animals by fecal microbiota transplantation (1), substantiating the causative and/or mediatory role of gut microbiota particularly in the context of diet-induced metabolic diseases.

The gut microbiota is dominated by the phyla Firmicutes and Bacteroidetes. Proteobacteria, Actinobacteria, Fusobacteria, Cyanobacteria, and Verrucomicrobia also occur but in much less abundance. The dysbiotic signatures in the gut microbiota associated with metabolic disease phenotypes include, remarkably, an increased ratio of Firmicutes to Bacteroidetes at phylum level (2, 3), an expansion of Proteobacteria (4, 5) and a reduced abundance of *Akkermansia* (6–9). Insulin-resistant phenotypes also exhibit a manifest proliferation of *Prevotella copri* and *Bacteroides vulgatus*, which elevate the circulating levels of branch chain amino acids (10). An obese microbiota was also found to associate with augmented serum glutamate levels due to the reduced abundance of *Bacteroides thetaiotaomicron* that converts glutamate (11). Moreover, an increased abundance of Proteobacteria and *Escherichia coli* with a reduction in the population of Firmicutes characterizes the gut microbiota associated with advanced fibrosis in human non-alcoholic fatty liver disease (NAFLD) (12). These dysbiotic microbiota configurations contribute to metabolic disorders by increasing energy harvest or by cross talking with the host immune, endocrine and nervous system *via* various nutrient sensing and signaling transduction mechanisms. Unraveling these mechanisms hence provides unique insights into the therapeutic opportunities for diabetes, obesity, and other metabolic diseases.

In light of the previous findings that have been efficiently summarized in several review articles (13–16), we would like to discuss the mechanistic basis of the emerging therapeutic strategies that target the host immune and nutrient-sensing pathways and to synthesize the most recent innovations and trends in the treatment of metabolic diseases.

## The Trialogue Between Nutritional Status, Gut Microbiota, and Immune System Reveals Novel Therapeutic Opportunities for Metabolic Diseases

**Figure 1:**
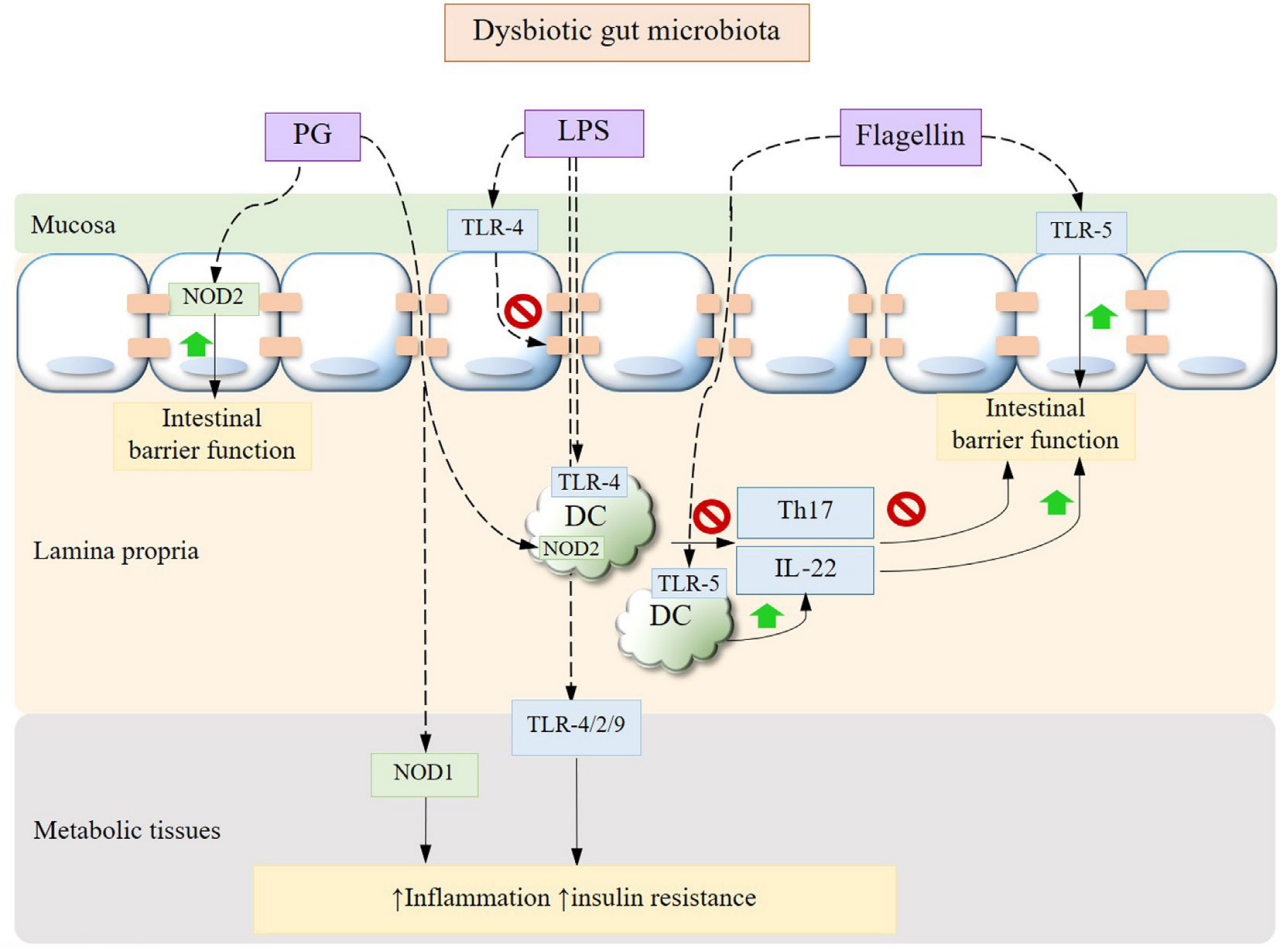
Gut microbiota dysbiosis-driven immune signaling pathways. Bacterial translocation occurs secondary to the mucosal epithelial barrier impairment driven by dysbiotic alterations in gut microbiota, leading to elevated circulating and tissue MAMPs such as LPS and PG. Bacterial LPS can disrupt the expression of epithelial tight junctions and, upon being translocated to peripheral tissues, trigger inflammation, and insulin resistance through toll-like receptors (TLRs). While PG induces tissue inflammation *via* NOD1, its recognition by NOD2 in intestinal epithelium confers protection against gut barrier dysfunction. The cross talk between APC and Th17 cells is also impaired under dysbiotic conditions with a decrease in IL-22. Adversely, the interactions between bacterial flagellin and intestinal epithelium or APC *via* TLR-5 improve gut barrier function. MAMPs, microbe-associated molecular patterns; LPS, lipopolysaccharide; PG, peptidoglycan; APC, antigen-presenting cells; IL, interleukin.

Metabolic diseases are characterized by a state of chronic subclinical inflammation in metabolic tissues such as liver, adipose, muscles, and pancreatic islets. The causative role of a dysbiotic gut microbiota in this inflammatory status by virtue of engaging diverse signaling transduction pathways and immune responses has been increasingly established in the past decade. In light of the increasingly unraveled trialogue between diet, gut microbiota, and the host immune system, a multitude of therapeutic approaches against metabolic diseases have emerged. One compelling set of mechanisms dictate the translocation of commensal bacteria and bacterial fragments toward metabolic tissues, where they trigger pro-inflammatory responses at the early onset of metabolic disorders. Evidence suggests that this translocation is promoted by a diet/microbiota-driven gut barrier impairment in dysbiotic conditions, thereby continuously fueling the host immune machinery that orchestrates the innate and adaptive arms (Figure 1).

High-fat diet (HFD) is known to induce a lipopolysaccharide (LPS)-enriched intestinal microbiota with a consequently elevated plasma concentration of LPS, which characterizes a state of metabolic endotoxemia (17). In the intestinal tract, LPS triggers the dysfunction of the mucosal barrier. Specifically, HFD has been shown to induce an increase in gut permeability by impairing the expression of tight junction proteins (17) *via* a mechanism that involves LPS-induced activation of the toll-like receptor (TLR)-4 pathways (18), while prebiotic carbohydrates treatment help reverse this impairment *via* a microbiota-driven modulation of endogenous proglucagon-derived peptide (GLP-2) by enteroendocrine L cells (19). HFD also reduces the mucus layer thickness, which however could be normalized upon administration of the mucin-producing *Akkermansia muciniphila* or prebiotics that restore its abundance *via* the endocannabinoids system that essentially regulate the gut barrier function (6). HFD has also been shown to cause a loss of intestinal T helper (Th) 17 cells putatively through an altered gut microbiota that interfere with the induction of Th17 cells by the antigen-presenting cells, resulting in diminished intestinal defense and integrity (20). Adoptive transfer of Th17 cells to obese mice shapes the gut microbiota to resemble that of a lean phenotype by increasing the ratio of Bacteroidetes to Firmicutes and the abundance of *Akkermansia*, contributing to improved metabolic profile to the recipients (21). Not surprisingly, HFD also impairs the induction of interleukin (IL)-22, which plays an essential role in eliciting antimicrobial immunity and maintaining mucosal barrier integrity within the intestine. Accordingly, promoting the production of IL-22 can be a potential intervention strategy to restore mucosal barrier integrity to reduce tissue inflammation and to improve metabolic profile (22). Moreover, it has been demonstrated that immunization with the HFD-associated ileum microbial extracts reversed partially gut microbiota dysbiosis and prevented hyperglycemia and insulin resistance in response to HFD likely by enhancing the proliferation of intestinal CD4 T cells (23). This underlines the potential of vaccination for preventing and managing T2DM.

The mechanism of bacterial translocation involves the host recognition receptors for the microbe-associated molecular patterns, including CD14 that binds bacterial LPS and the peptidoglycan (PG) sensor NOD1 (24). Of note, sensing of PG by NOD2 confers, however, protection in HFD-induced metabolic disease against gut barrier impairments and the microbiota-driven tissue inflammation (25, 26). It follows that manipulation of intestinal bacterial adherence, bacterial translocation and receptors for bacterial fragments could be promising strategies to prevent or revert the incidence of metabolic disorders. For example, treatments with probiotics, such as *Bifidobacteria* (24, 27) and *Akkermansia* (28), have been demonstrated to be efficacious against the adherence and translocation of mucosal bacteria, tissue inflammation, and insulin resistance by modulating the gut microbial structure. The administration of polyphenols as prebiotics also confers similar protective effects in diet-induced metabolic syndrome by increasing the abundance of *Akkermansia* (29). The role of bacterial muramyl dipeptide as a postbiotic has also been documented in reducing inflammation and promoting insulin signaling in the state of metabolic endotoxemia, glycemia, and obesity *via* a pathway that involves NOD2 (30).

Upon being translocated to metabolic tissues, LPS can elicit pro-inflammatory responses *via* pathways mediated primarily by TLR-4 (31) with the involvement of TLR-2 (32) and TLR-9 (33), diminishing insulin signaling and increasing adiposity in the state of obesity and T2DM. This cross talk is mediated by gut microbiota as shown by the abrogation of these metabolic endotoxemia-induced phenotypes along with a decrease in inflammation markers and intestinal permeability after an antibiotic treatment (17). Anti-inflammatory agents, such as 5-aminosalicylic acid, therefore prove to be efficacious in restoring gut barrier and reducing tissue microbiota dysbiosis and inflammation in metabolic syndrome (34). The attenuation in metabolic endotoxemia also serves as one mechanism that contributes to the resolution of insulin resistance and T2DM after Roux-en-Y gastric bypass (RYGB) (35). The RYGB-associated, markedly diminished endogenous (i.e., microbiota derived), LPS highlights the role of a modulated gut microbiota as a mediator of metabolic conditions (36). Gut microbial homeostasis is also subject to regulation by intestinal inflammasomes, the loss of which can induce a dysbiotic microbiome and exacerbate metabolic endotoxemia, as observed in the liver, leading to the development of hallmark features of NAFLD including increased hepatic steatosis, inflammation, and insulin resistance (33). Metabolic endotoxemia also induces endoplasmic reticulum stress and concomitantly the activity of histone acetyltransferase p300, which acetylates insulin receptor substrate, prevents its association with the insulin receptor and hence impairs insulin signaling (37). Likewise, TLR-5-dependent signaling regulates the gut microbial ecology by sensing bacterial flagellin, conferring protection against metabolic diseases (38, 39). Myeloid differentiation primary response 88 (MyD88) is an important signaling component of TLR pathways. It is worth noting that tissue-specific MyD88 deficiency confers distinct phenotypes. Specifically, intestinal specific deletion of MyD88 putatively serves to normalize the gut barrier function and increase energy expenditure during HFD feeding in a microbiota-dependent manner, leading to reduced adiposity and inflammation and an improved glucose homeostasis (40). Conversely, hepatocyte-specific deletion of MyD88 disrupts glucose and lipid homeostasis presumably by inducing changes in the related gene expression, bile acid signaling, and in the gut microbiota configuration (41). Deletion of MyD88 has also been demonstrated to reduce the incidence of spontaneous type I diabetes by inducing transmissible changes in the gut microbiota (42). These findings support the role of MyD88 as a pharmacological target for metabolic disease, though it multifaceted functions warrant further investigation.

## Nutrient-Sensing Mechanisms are Key Metabolic Mediators of Host Responses to Gut Microbiota Alterations

**Figure 2:**
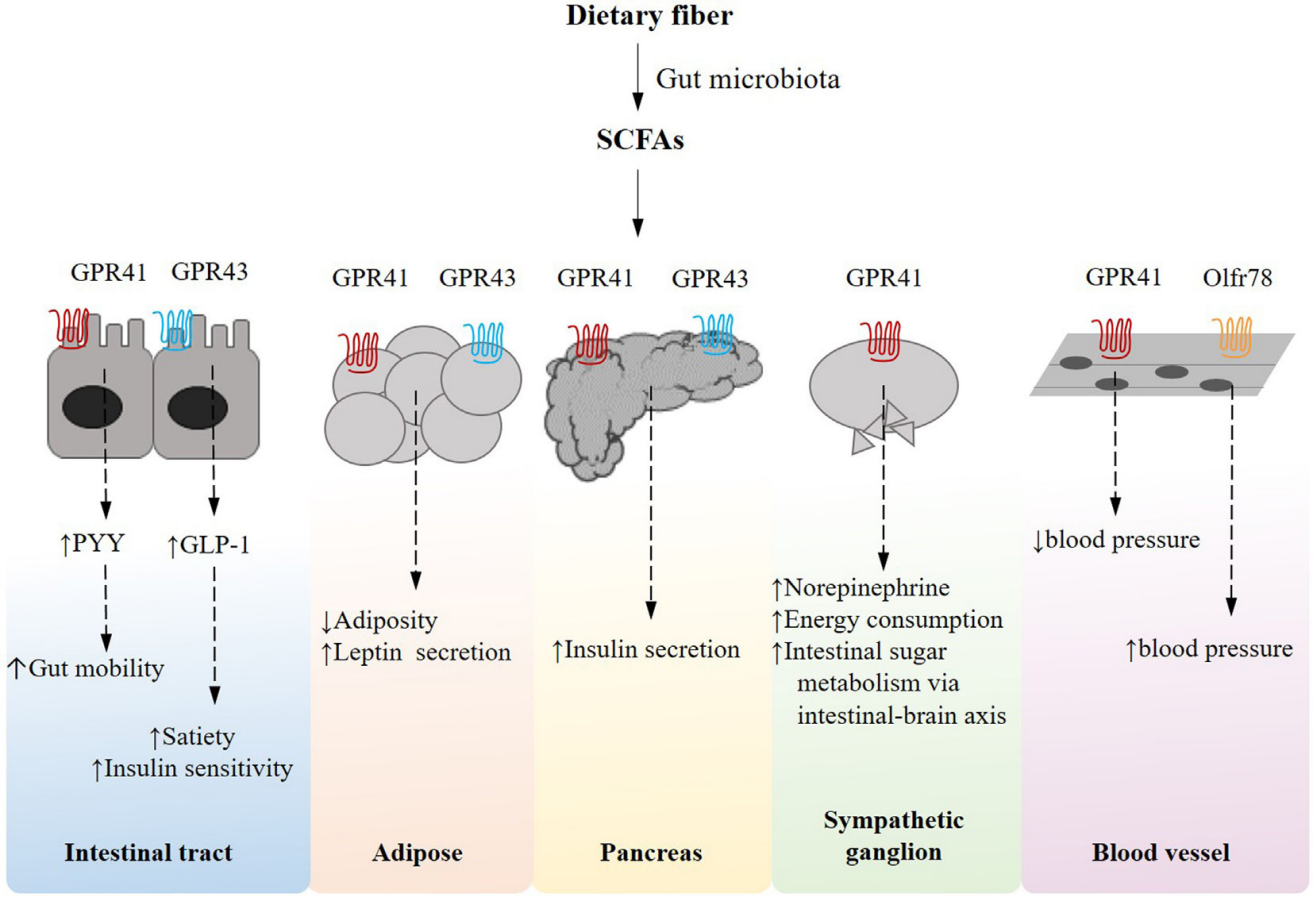
Short-chain fatty acid (SCFA)-receptor-mediated pathways and their effects on host energy metabolism in peripheral tissues. Gut microbes can ferment dietary fiber into SCFAs, which induce an array of G-protein coupled receptor-mediated signaling pathways that are essentially implicated in host energy homeostasis in multiple tissues.

It has been increasingly established that in the metabolic machinery G-protein coupled receptor (GPCR)-mediated nutrient sensing serves as essential mechanisms that coordinate host responses to dietary and endogenous nutrients, gaining significant interest as a putative therapeutic target for metabolic diseases. A distinct subset of the nutrient-sensing receptors is specifically activated by free fatty acids (FFAs) of various chain lengths. In particular, short-chain fatty acids (SCFAs), including primarily acetate, propionate, and butyrate, are the most abundant microbial metabolites derived from the otherwise indigestible dietary polysaccharides and the natural ligands for fatty acid receptors such as GPR41/FFAR3, GPR43/FFAR2, GPR109A, and Olfr78 (Figure 2). Activation of the GPR41 expressed on the enteroendocrine cells is reported to stimulate the secretion of the gut hormone peptide YY, which functions to reduce energy intake (43, 44), while sensing of SCFAs by GPR43 triggers the production of glucagon-like peptide-1 (GLP-1), which is known to decrease gastric emptying rate and improve glucose-induced insulin secretion (44–46). GPR41 and GPR43 also occur on intestinal epithelial cells, upon stimulation by SCFAs, playing protective role against intestinal inflammation by inducing inflammasome activation (47). A propionate-dependent signaling of GPR41 is also involved in a gut–brain neural circuit that regulates intestinal gluconeogenesis, a substantial mechanism that ensures metabolic homeostasis (48). In adipose tissues, SCFA–GPR41 interaction correlates with the circulating level of leptin, thereby regulating food intake (49), while GPR43 mediates insulin signaling and adipogenesis (50). SCFAs, acting through GPR41 expressed in neurons of the superior cervical ganglion, also contribute to increased energy expenditure by increasing sympathetic nervous system activity (51). SCFAs, in addition, have been suggested to regulate the proliferation of pancreatic β cells and insulin biosynthesis *via* GPR41 or GPR43 (52–55). GPR109A is a receptor for niacin but also responds to butyrate, contributing to reduced colonic inflammation and homeostatic lipid metabolism in adipose tissues (56). On the other hand, SCFAs play a role in blood pressure regulation *via* the receptors in blood vessels. Specifically, the activation of GPR41 has been observed to reduce blood pressure (57), whereas an olfactory receptor Olfr78, upon stimulation by acetate and propionate, leads to an increased blood pressure (58). It is also worth noting that endogenously derived SCFAs confer an epigenetic mechanism whereby chromatin structure and cell fate allocation respond to gut microbiota and diet (21, 59, 60). Taken together, it follows that nutrient-sensing receptors could be therapeutic targets and their agonism a potent strategy that enhances insulin signaling, energy homeostasis and intestinal function, highlighting the significance of dietary microbiota-accessible carbohydrates (61) and the development of new agonists for the treatment of metabolic diseases. Despite the evidence supportive of the beneficial role of dietary SCFAs in maintaining metabolic homeostasis, controversially, for instance, a microbiota-dependent excessive accumulation of SCFAs driven by immunodeficiency of TLR-5 signaling has been documented to promote low-grade inflammation, augmenting hepatic *de novo* lipogenesis and insulin resistance (62). This discrepancy warrants further studies on the role of SCFAs and their receptors in the context of disease model, dose, and tissue dependence.

In addition to the SCFA-dependent GPCRs, GPR119 recognizes long-chain fatty acids and derivatives as endogenous ligands. It is worth mentioning that GPR119 is a validated therapeutic target for T2DM in that it essentially mediates glucose-induced insulin secretion and intestinal secretion of GLP-1 and glucose-dependent insulinotropic peptide (63–65). Recently, Cohen et al. (66) identified striking structural and functional similarities between human ligands for GPR119 and their microbiota-encoded counterparts, proposing a new perspective on antidiabetic agents that dictates genetic modulation of microbiota-derived metabolites to elicit desirable host responses.

Nuclear farnesoid X receptor (FXR)-mediated bile acid signaling also represents a nutrient-sensing mechanism that regulates glucose and lipid metabolism. Gut microbiota plays a pivotal role in the biotransformation and thereby the signaling of bile acids. It has been recently reported that the obese phenotype is associated with a gut microbiota that promotes deconjugation of bile acids, resulting in increased hepatic steatosis and weight gain *via* the activation of FXR (67, 68), which may bear implications for treating metabolic diseases.

## Concluding Remarks

As the dysbiosis of gut microbiota is increasingly appreciated as a mechanism accounting for metabolic diseases, the growing understanding of microbiota-driven immune and metabolic responses has begun to reveal potential interventional targets, sustaining the development of new therapeutic strategies by dietary and/or pharmacological means. These progresses highlight the therapeutic significance of (1) dietary intervention (e.g., prebiotics, probiotics, and postbiotics) and fecal microbiota transplantation that tackle microbiota dysbiosis and (2) manipulation of the key players in the intestinal integrity and function, bacterial recognition and translocation, tissue inflammation, and host nutrient-sensing mechanisms by using such as receptor agonists/antagonists and anti-inflammatory agents; in particular, recent reports about vaccination and genetic modulation of the gut microbial metabolites further add to our knowledge of the therapeutics of metabolic diseases (Table 1).

**Table 1:** Potential therapeutic targets and strategies revealed by the mechanistic basis of the pathophysiology of metabolic diseases.

Potential therapeutic target and strategy	Reference
Prebiotics (e.g., carbohydrates and polyphenols)	(6, 19, 29)
Probiotics (e.g., *Akkermansia muciniphila* and *Bifidobacteria*)	(6, 24, 27, 28)
Postbiotics (e.g., bacterial muramyl dipeptide)	(30)
Adoptive transfer of T helper 17 cells	(20)
Anti-inflammatory agents (e.g., 5-aminosalicylic acid)	(34)
Immunization with the high-fat diet-associated ileum microbial extracts	(23)
NOD1 antagonists	(24)
NOD2 agonists	(25, 26)
Myd88 agonists or antagonists	(40–42)
Toll-like receptor-5 agonists	(38, 39)
Improving gut microbial production of short-chain fatty acids	(21, 59, 60)
Farnesoid X receptor antagonists	(67, 68)
GPR41 agonists	(44, 47, 48, 51)
GPR43 agonists	(44, 45, 50)
GPR109A agonists	(56)
Olfr78 antagonists	(58)
GPR119 agonists	(63–65)
Genetic modulation of microbiota-derived metabolites	(66)

## Author Contributions

XL and KW wrote the paper. IK supervised and wrote the paper.

## Conflict of Interest Statement

The authors declare that the research was conducted in the absence of any commercial or financial relationships that could be construed as a potential conflict of interest.
